# Pulmonary atresia with a ventricular septal defect and left pulmonary artery discontinuity: a case report 

**DOI:** 10.1186/s13256-021-02750-4

**Published:** 2021-04-01

**Authors:** Hyun-Hwa Cha, Hae Min Kim, Won Joon Seong

**Affiliations:** Department of Obstetrics and Gynecology, Kyungpook National University Hospital, School of Medicine, Kyungpook National University, 807 Hogukro, Buk-gu, Daegu, 41404 Republic of Korea

**Keywords:** Fetal disease, Pulmonary artery, Echocardiography

## Abstract

**Background:**

Unilateral pulmonary artery discontinuity is a rare malformation that is associated with other intracardiac abnormalities. Cases accompanied by other cardiac abnormalities are often missed on prenatal echocardiography. The prenatal diagnosis of isolated unilateral pulmonary artery discontinuity can also be delayed. However, undiagnosed this malformation would have an effect on further prognosis. We report our case of a prenatal diagnosis of pulmonary atresia with ventricular septal defect and left pulmonary artery discontinuity.

**Case presentation:**

A 33-year-old Asian woman visited our institution at 24 weeks of gestation because of suspected fetal congenital heart disease. Fetal echocardiography revealed a small atretic main pulmonary artery giving rise to the right pulmonary artery without bifurcation and the left pulmonary artery arising from the ductus arteriosus originating from the left subclavian artery. The neonate was delivered by cesarean section at 37^6/7^ weeks of gestation. Postnatal echocardiography and multidetector computed tomography showed a right aortic arch, with the small right pulmonary artery originating from the atretic main pulmonary artery and the left pulmonary artery originating from the left subclavian artery. Patency of the ductus arteriosus from the left subclavian artery was maintained with prostaglandin E1. Right ventricular outflow tract reconstruction and pulmonary angioplasty with Gore-Tex graft patch was performed 25th day after birth. Unfortunately, the neonate died because of right heart failure 8 days postoperation.

**Conclusion:**

There is a possibility that both pulmonary arteries do not arise from the same great artery (main pulmonary artery or common arterial trunk). Therefore, clinicians should check the origin of both pulmonary arteries.

## Background

Unilateral pulmonary artery discontinuity (UPAD) is a rare form of congenital heart disease (CHD) that is usually accompanied by other cardiac anomalies, though it may occur as an isolated form [1]. When it is complicated with other CHDs, the precise prenatal diagnosis may be obscured. In contrast, isolated UPAD is often missed in the prenatal, neonatal, or infantile period. Therefore, it is detected only after patients develop clinical symptoms such as exercise intolerance, dyspnea, chest pain, hemoptysis, and recurrent pulmonary infections, throughout infancy and adolescence [2]. We report a case of pulmonary atresia with ventricular septal defect (PAVSD) with LPA discontinuity.

## Case presentation

**Fig. 1.**
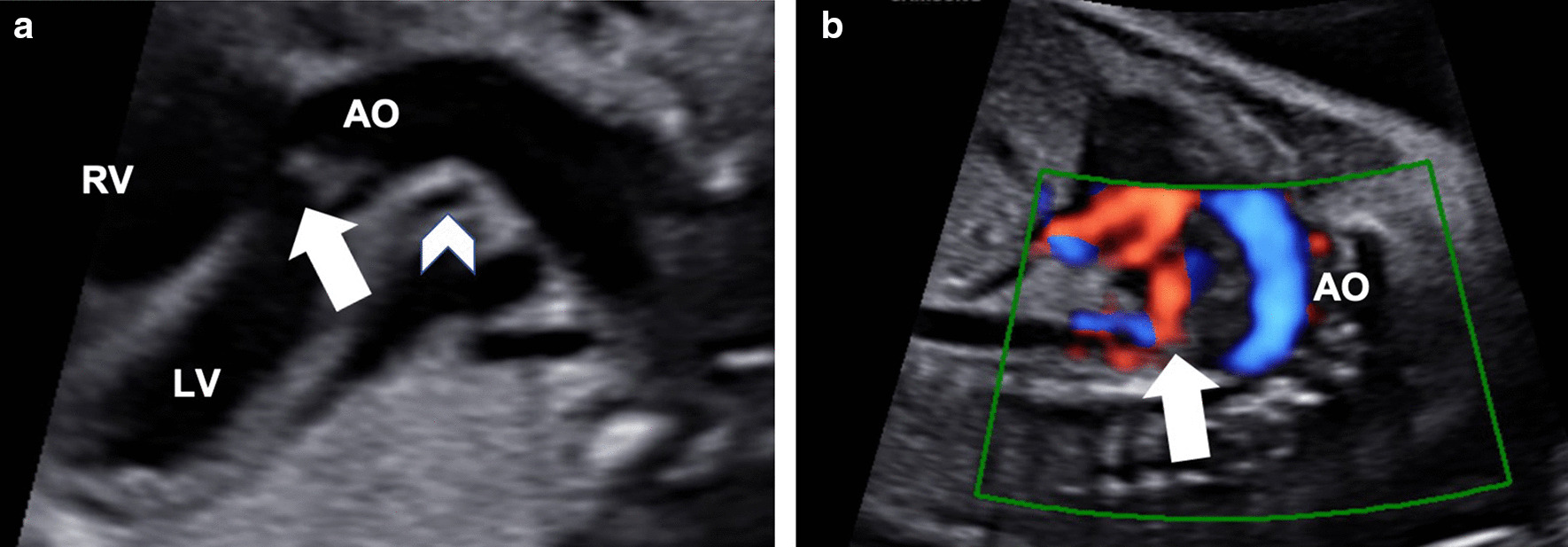
Prenatal echocardiogram showing a large ventricular septal defect, an overriding aorta (arrow), and an atretic main pulmonary artery (arrowhead) (**a**) and multiple aortopulmonary collateral arteries (**b**)

**Fig. 2.**
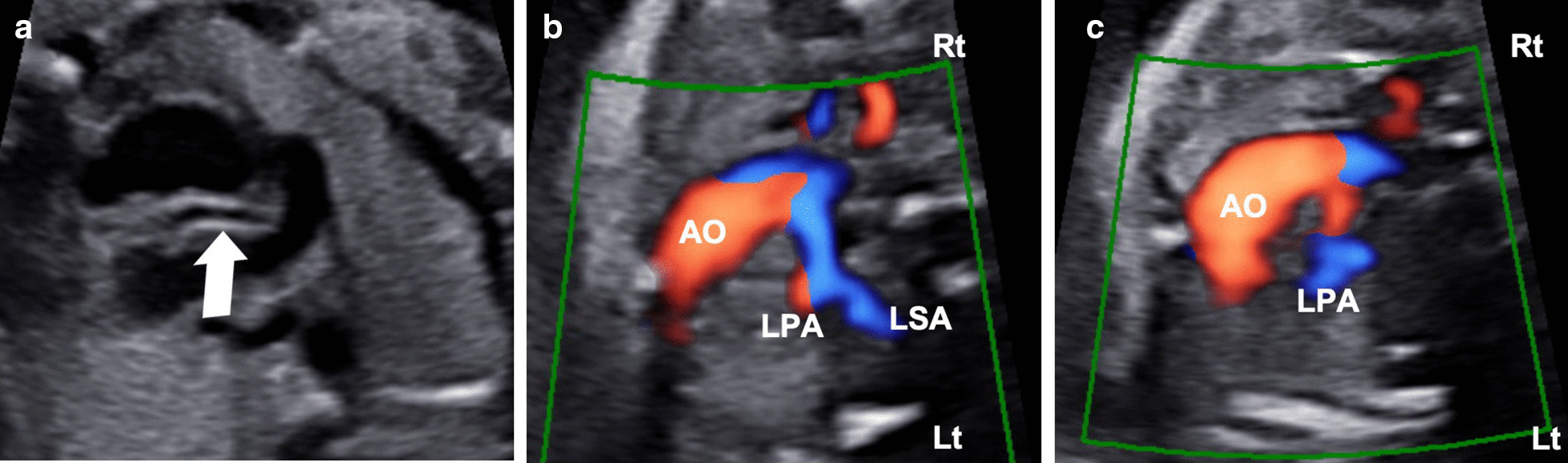
Prenatal echocardiogram showing an atretic main pulmonary artery giving rise to the right pulmonary artery (arrow) (**a**), left subclavian artery (LSA) from aortic arch (**b**), and left pulmonary artery (LPA) arising from the left subclavian artery (**c**)

**Fig. 3.**
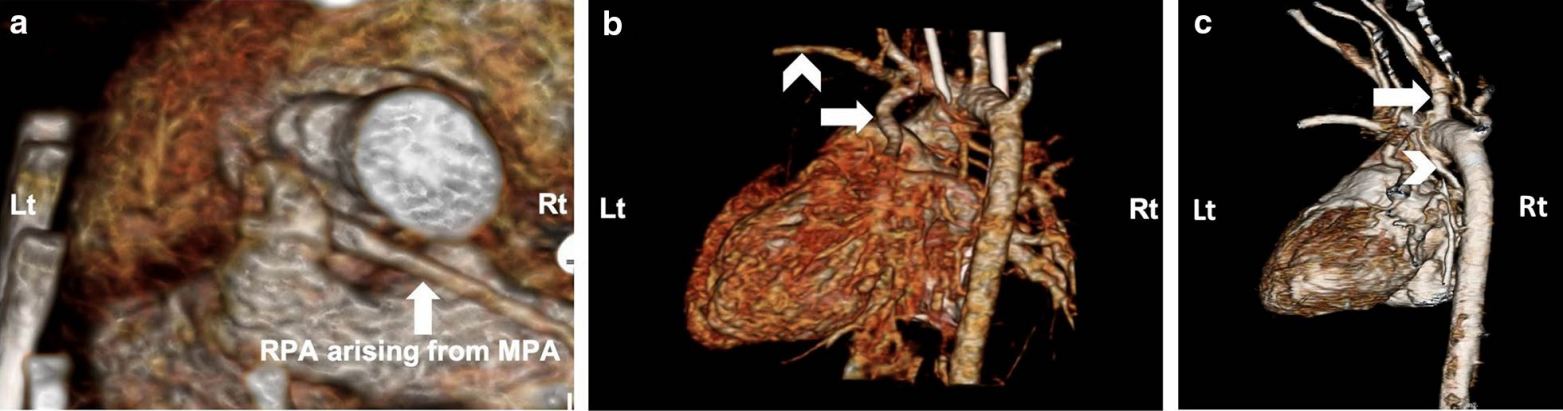
Postnatal multidetector computed tomography image showing the posterior aspect of the neonate. Also shown are the right pulmonary artery (white arrow) arising from the atretic main pulmonary artery (**a**) and the left pulmonary artery (white arrow) arising from the left subclavian artery (arrowhead) (**b**). Multiple aortopulmonary collateral arteries (MAPCAs, arrowhead) from right common carotid artery (white arrow) supply the right lung (**c**)

A 33-year-old pregnant Asian woman (gravida 1, para 0) was referred to our hospital at 24 weeks of gestation because of a suspected fetal heart anomaly on routine obstetric ultrasonography. The results of her prenatal laboratory tests were normal. Fetal echocardiography revealed a large ventricular septal defect (VSD) measuring 5 mm with a large overriding aorta (Fig. 1a). We also observed multiple major aortopulmonary collateral arteries (Fig. 1b). Therefore, the initial prenatal diagnosis was pulmonary atresia with VSD (PAVSD). A very atretic main pulmonary artery (MPA) can be seen in Fig. 1a retrospectively; however, we were not able to detect this artery at that time. The subsequent fetal echocardiography, which we performed at 26 weeks of gestation, revealed a highly atretic MPA from the right ventricle (RV) giving rise to the right pulmonary artery (RPA), without bifurcation (Fig. 2a). Instead of the bifurcation of the MPA, the left pulmonary artery (LPA) originated from the left subclavian artery (LSA; Fig. 2b, c). The echogenicity of the thymus was not definitive on prenatal echocardiography. On the basis of these findings, the fetus was diagnosed as having PAVSD with left PAD and 22q11.2 deletion syndrome. Considering the gestational age at diagnosis, we decided to postpone the genetic study to after birth. A female neonate was delivered by elective cesarean section at 37^6/7^ weeks of gestation for the timed delivery, with a body weight of 2740 g, Apgar score of 8/9 points, heartbeat of 155 beats per minute, respiratory rate of 44 breaths per minute, blood pressure of 71/38 mmHg, and SpO_2_ of 88%. Multidetector computed tomography (MDCT) revealed a right-sided aortic arch, with the left-sided ductus arteriosus (DA) originating from the LSA and MAPCA. It also revealed a narrow RPA (2.7 mm) connecting with the MPA (2.7 mm), without connection with the LPA (2.7 mm size). The LPA originated from the left-sided DA originating from the LSA. Three-dimensional MDCT images showed the posterior aspect of the heart of the affected neonate (Fig. 3a–c).

The neonate was assisted using the noninvasive continuous positive airway pressure (CPAP) method because of chest retraction and tachycardia. To maintain the patency of the DA, administration of prostaglandin E1 α-cyclodextrin clathrate (PGE1-CD; Eglandin) 5 ng/(kg·min) was initiated immediately after birth, and the dosage was adjusted to between 3 and 5 ng/(kg·min), targeting 85% of the SpO_2_. Meanwhile, postnatal multiplex ligation-dependent probe amplification (MLPA) revealed that the neonate’s condition was complicated by 22q11.2 deletion syndrome.

The general condition of the neonate remained stable; therefore, we attempted to wean the neonate from CPAP to high-flow nasal cannula (3 L) and tapered the PGE1-CD dosage to 1 ng/(kg·min). However, on the 15th day after birth, the SpO_2_ fluctuated and required assist-control mandatory ventilation with intubation. After the neonate stabilized, right ventricular outflow tract (RVOT) reconstruction and pulmonary artery reimplantation were performed on the 25th day after birth. Postoperative echocardiography revealed a RVOT without turbulent flow and bilateral pulmonary arteries measuring 4.2 mm (left) and 3.3 mm (right). However, the SpO_2_ fluctuated, and hemoptysis requiring full sedation occurred on the seventh day after the operation. A subsequent echocardiography revealed decreased blood flow in the right ventricle and RPA with pericardial effusion; thus, right heart failure was suspected. We started milrinone administration to augment ventricular contractility and decrease the afterload. However, the neonate died because of right heart failure.

## Discussion

UPAD is a rare congenital malformation and usually presents as an absence of proximal unilateral pulmonary artery on prenatal echocardiography; therefore, it has also been referred to as unilateral absence of the pulmonary artery [1]. As the distal segment of the affected pulmonary artery actually exists, UPAD is a more accurate term to describe this malformation [1]. It also should be distinguished from the other rare form of CHD, hemitruncus arteriosus, which is defined as an anomalous origin of one of the branch pulmonary arteries (PAs) from the aorta and a normal origin of other PAs from the RVOT [2]. If untreated, it results in a large left-to-right shunt, with the whole cardiac output from the right ventricle directed to the unaffected lung while the other lung receives blood at a systemic pressure from the systemic aorta; its 1-year survival rate has been reported to be <30% [2, 3].

Unlike patients with hemitruncus arteriosus, those with UPAD receive blood supply to one lung from ductus-like collateral vessels, not the systemic aorta, and it is often associated with other CHDs, especially tetralogy of Fallot (TOF) [2]. Although its pathophysiology is not fully understood, most studies on UPAD have described that a failure in the connection of the sixth aortic arch with the pulmonary trunk results in this developmental anomaly [1, 4]. Clinical presentations vary depending on the affected site [1, 4, 5]. In the case of LPAD, 75% of patients have an associated congenital heart disease, including TOF, right-sided aortic arch, septal defect, or persistent DA [1, 5, 6]. By contrast, most patients with RPA discontinuity have the isolated form without other intracardiac anatomies. The affected pulmonary artery is on the side opposite the aortic arch [6]. As known previously, in this case, the LPA was affected, and the neonate showed a right aortic arch. This implies that confirmation of the aortic arch location is of great clinical significance.

As mentioned above, the initial diagnosis was PAVSD only. In the case of PAVSD, the initial maintenance of DA is also important because the pulmonary circulation is dependent on DA. However, the atretic MPA was not dependent on DA in the present case. Instead, right lung was supplied by MAPCA rather than by atretic MPA. If PGE1 was administered for a certain period to maintain DA without prenatal detection of UPAD in our case, it would be associated with a decrease in the size of the right PA, resulting in PA size discrepancy. Therefore, we think the detection of UPAD before surgery would help to decide the timing and planning of the surgery. Meanwhile, an isolated form of UPAD (usually affecting the right PA) could be missed during prenatal care. Diagnosis is often delayed in patients with pulmonary hypertension, recurrent pulmonary infections, congestive heart failure, and hemoptysis [4]. Prenatal detection is important because it aids in the prompt initiation of PGE1 administration to ensure early rehabilitation of the affected lung [7].

No consensus has been reached regarding the treatment for UPAD. In cases complicated by other CHDs, treatment would depend on major cardiac abnormalities. In our case, the prenatal detection of UPAD in addition to PAVSD allowed the pediatric cardiologist to make a precise operative plan. Our pediatric cardiologist knew the size difference between the LPA and the MPA, which made it possible to prepare the Gore-Tex graft patch for pulmonary angioplasty. In cases of the isolated form of UPAD, early intervention for UPAD has been supported owing to the concern for regression of the affected PA after DA closure. However, the operation timing should be determined on the basis of many other clinical situations, including the neonatal condition or birth weight [7]. If early intervention is unavailable, administration of PGE1 is usually required. In cases of delayed diagnosis made in the adolescent or adult period, lobectomy and ligation of the affected PA are required [5].

## Conclusion

UPAD is relatively rare, but when undetected, it could affect neonatal prognosis. Therefore, clinicians should examine the route of both pulmonary arteries, regardless of the existence of other intracardiac abnormalities.

## Data Availability

All data generated or analyzed during this study are included in this manuscript. The datasets during and/or analyzed during the current study are available from the corresponding author on reasonable request.

## Abbreviations

UPAD: Unilateral pulmonary artery discontinuity
PAVSD: Pulmonary atresia with ventricular septal defect
CHD: Congenital heart disease
MPA: Main pulmonary artery
RPA: Right pulmonary artery
LPA: Left pulmonary artery
DA: Ductus arteriosus
LSA: Left subclavian artery
MDCT: Multidetector computed tomography
RVOT: Right ventricular outflow tract
TOF: Tetralogy of Fallot
